# The role of amyloid β in the pathological mechanism of GNE myopathy

**DOI:** 10.1007/s10072-022-06301-7

**Published:** 2022-07-29

**Authors:** Tongtong Zhang, Ren Shang, Jing Miao

**Affiliations:** Department of Neurology, The First Hospital of Jilin University, Changchun, China

**Keywords:** Amyloid β, Autophagy, GNE myopathy, Mitophagy, Sialic acid, Muscle atrophy

## Abstract

GNE myopathy is a hereditary muscle disorder characterized by muscle atrophy and weakness initially involving the lower distal extremities. The treatment of GNE myopathy mainly focuses on a sialic acid deficiency caused by a mutation in the *GNE* gene, but it has not achieved the expected effect. The main pathological features of GNE myopathy are myofiber atrophy and rimmed vacuoles, including accumulation of amyloid β, which is mainly found in atrophic muscle fibers. Although the role of amyloid β and other misfolded proteins on the nervous system has been widely recognized, the cause and process of the formation of amyloid β in the pathological process of GNE myopathy are unclear. In addition, amyloid β has been reported to be linked to quality control mechanisms of proteins, such as molecular chaperones, the ubiquitin–proteasome system, and the autophagy-lysosome system. Herein, we summarize the possible reasons for amyloid β deposition and illustrate amyloid β-mediated events in the cells and their role in muscle atrophy in GNE myopathy. This review represents an overview of amyloid β and GNE myopathy that could help identify a potential mechanism and thereby a plausible therapeutic for the disease.

## Introduction

GNE myopathy is an autosomal recessive disease known as distal myopathy with rimmed vacuoles (DMRV), hereditary inclusion–body myopathy, or Nonaka myopathy [1]. The onset of GNE myopathy usually occurs in early adulthood as weakness of the distal tibial muscle in the lower limbs. In later stages, the proximal muscles, respiratory muscles, and myocardium are also affected with relative sparing of the quadriceps [2]. GNE myopathy is caused by a mutation in the *GNE* gene [3], which encodes a bifunctional enzyme with UDP-N-acetylglucosamine 2-epimerase activity in the amino-terminal region and N-acetylmannosamine kinase activity in the carboxy-terminal region. It is expressed in different tissues and plays a critical role in sialic acid (SA) biosynthesis [4]. Over 201 mutations in *GNE*, spanning the epimerase and kinase domains and allosteric region can lead to a disorder of SA synthesis. *GNE*-knockout mouse embryos can hardly survive; no human carriers with two nonsense or frameshift mutations have been found to date, suggesting that SA is important during embryonic or early development [5]. Homozygous mutations in the kinase domain can cause the typical clinical features of GNE myopathy, but compound heterozygous *GNE* mutations exhibit different clinical features and disease progression. Patients with kinase domain mutations progress faster than those with heterozygous mutations in terms of joint function [6]. It is suggested that different variants have different effects on function [7]. The spectrum of diseases caused by *GNE* mutations has continued to increase in recent years. Originally, *GNE* mutations were implicated in the pathophysiology of amyotrophic lateral sclerosis [8]. There are also *GNE* mutations associated with the Charcot-Marie-Tooth type 2 phenotype [9]. Recent studies have found that *GNE* mutations affect both muscles and nerves, and multiple patients have evidence of axonal motor nerve involvement [10, 11]. Patients with GNE myopathy have extramuscular manifestations of idiopathic thrombocytopenia, cardiac damage, sleep apnea syndrome, and respiratory dysfunction, the etiologies of which are unknown [12]. The diversity of clinical phenotypes reminds us that we must investigate other GNE enzymes’ roles. Fortunately, human pluripotent stem cells have been used to establish isogenic GNE myopathy disease models which can explore gene-phenotype relationships and develop drugs [13]. Although the exact pathological mechanism of GNE myopathy is unknown, aberrant protein sialylation promoting the progression of GNE myopathy cannot be ignored [14, 15].

The primary pathological manifestation of GNE myopathy is the presence of rimmed vacuoles (RVs) in atrophic muscle fibers [16, 17]. Most RVs are round or oval in the center of atrophic muscle fibers surrounded by basophilic granules. RVs appear empty in pathological staining due to the detachment of red-colored granules during staining, and these granules are formed by protein aggregates, such as amyloid β (Aβ) and tau [18]. Malicdan et al. considered that RVs are empty spaces surrounded by an aggregation of autophagic vacuoles and have high acid phosphatase activity, lysosomal marker reactivity, and multilamellar bodies [17, 19]. Under electron microscopy, the RV region appears to have an autophagosomal structure, which contains degradation products from the membrane, cytoplasm, and mitochondria. These phenomena show that the RV area involves a continual process of autophagy [20]. Li et al. observed that the amyloid precursor protein (APP) exists in different muscle fibers in various forms. In atrophic muscle fibers, it accumulates in patches of a substance that is probably Aβ. These abnormal APP deposits are presumed to be an early stage of Aβ deposition in roughly normal muscle fibers [21]. In mouse models, Aβ accumulation begins from 32 to 34 weeks of age when there is no RV formation in the muscles or apparent muscle atrophy. Aβ deposition and Aβ-like structures in atrophic muscle fibers before RV formation suggest that abnormal protein accumulation is an upstream event contributing to the pathogenic cascade of GNE myopathy [17, 22]. This evidence shows that the Aβ peptide is the key factor in GNE pathogenesis. Furthermore, Aβ can disrupt Ca^2+^ homeostasis and mitochondrial function and activate the endoplasmic reticulum (ER) stress response, which triggers the ubiquitin–proteasome and autophagolysosome systems.

In this study, we provide an overview of the clinical presentation of GNE myopathy. Importantly, we review the recent advances in understanding the relationship between Aβ and the pathological mechanism of GNE diseases emphasizing molecular chaperones, autophagy, and cell apoptosis. In addition, we summarize molecular mechanisms which may lead to muscle atrophy. This review offers new insights that may contribute to future therapies for GNE myopathy.

### Clinical manifestations

The most prominent clinical manifestation of GNE myopathy is the weakness of the distal lower extremities, in which tibialis anterior muscle is most seriously involved. The typical clinical presentation is foot drop, and due to tissue-specific expression of SA, GNE myopathy may involve multiple systems. Cardiomyopathy in patients with GNE myopathy has been reported in the past [23]. In addition, pregnancy and childbirth are important for the progression of the disease [24]. In a cohort study of patients with GNE myopathy, Jang et al. found a potential effect of GNE on platelet survival [25]. Numerous cases of *GNE* mutations leading to thrombocytopenia with or without muscle weakness have been reported [26–28]. The mechanism relates to Ashwell-Morell receptor recognition and classical and lectin pathway activation [29]. Beecher et al. found that platelet-associated immunoglobulin G (PA-IgG) frequently appeared in GNE myopathy through a cohort study. PA-IgG positivity is associated with reduced muscle strength, which has the potential to be used as a biomarker to assess the severity of GNE myopathy [30]. In addition to thrombocytopenia, Smolag et al. reported hyposialylation of leukocytes and erythrocyte in patients with *GNE* mutations, along with mild neutropenia and increased erythrocyte lysis. This may be because SA affects complement factor H (FH), which activates classical and lectin pathways. This eventually leads to hemolysis and complement activation [31]. Respiratory muscles may be involved in the later stages of the disease [32, 33] development. In addition, sleep apnea is an important complication of GNE myopathy. It is unclear whether the pathogenesis is obstructive or central [34]. Muscle magnetic resonance imaging (MRI) has demonstrated patterns of muscle involvement in GNE myopathy. At the early stage of the disease, the biceps femoris short head muscles of both lower limbs have a serious fatty-fibrous replacement. The anterior tibialis and toe dorsiflexors are also affected. However, the only surviving muscle in the late stages is not the entire quadriceps but the vastus lateralis [35]. Early damage of upper limb muscles is limited to the serratus anterior, subscapularis, trapezius, and pectoralis minor. However, the duration and severity of muscle damage in the upper limbs are less than those in the lower limbs. Paraspinal muscle involvement continues throughout the course of the disease; cranial muscles, including the tongue, were spared in all scans [36]. Gidaro and colleagues found that quantitative nuclear MRI (qNMRI) can detect significant muscle changes in patients with GNE myopathy within 1 year (i.e., change in fat percentage and contractile cross-sectional area). qNMRI indices showed a strong correlation with muscle strength [37]. Importantly, Liu et al. found that muscle involvement and disease severity can be monitored by skeletal muscle MRI and proton magnetic resonance spectroscopy (^1^H-MRS) [38]. In addition to muscle MRI, attempts have been made to use lectin staining to evaluate the change in sarcolemmal sialylation in human and mouse GNE myopathy muscle sections [39–41]. This assay can be used in phase II clinical trials of ManNAc [42]. Meanwhile, Sattler et al. compared the degree of myotube differentiation in patients with GNE myopathy with their unaffected members in search of biomarkers to evaluate SA therapy [43].

### Aβ in GNE myopathy

The Aβ peptide is a 39–43 amino acid residue peptide, a regular secretion product of the metabolism of transmembrane protein APP, which is mainly processed in two ways: amyloidosis and non-amyloidosis [44]. Through the amyloid pathway, APP is broken down into Aβ by β- and γ-secretase. Although abnormal Aβ deposition has been found in GNE myopathy, whether APP is abnormal in GNE myopathy is still unknown. Aβ peptides can form homodimers of different sizes and conformations or oligomers with β-pleated sheet structures [45]. The peptide assembly into oligomers lead to gain or loss of function. Aβ polypeptides are hydrophobic and tend to assemble to form aggregates. The most common form is the Aβ1-40 subtype, followed by Aβ1-42 [46–48]. Kayed and Lasagna-Reeves demonstrated that after being incorporated into the membrane, the Aβ conformation changes, resulting in aggregation on membranes [49]. Aβ1-42 is more cytotoxic than Aβ1-40, and one of the main hypotheses behind the toxic effects of Aβ is the membrane perturbation induced by Aβ through membrane fluidity, amyloid peptide channel formation, free radical production, and lipid peroxidation [50, 51]. Aβ oligomers have been shown to directly enter the membrane and form a porous structure [52] called “amyloid channels” that are selectively permeable to Ca^2+^, leading to a rapid increase in Ca^2+^ levels and disrupting Ca^2+^ homeostasis in cells. Abnormal protein deposits can combine with mitochondrial-related proteins, hinder the entry of mitochondrial proteins related to oxidative phosphorylation and affect mitochondrial function leading to the activation of the apoptotic signaling cascade [53, 54]. Once the mitochondrial network accumulates damage, followed by oxidative stress, the mitochondrial membrane potential will be affected, triggering PINK1 aggregation to initiate mitochondrial autophagy [55]. Also, the autophagy signal mediated by PINK1 contributes to Aβ degradation and clearance; these functions were interrelated in a transgenic mouse model overexpressing human APP mutants [56]. All these intracellular events caused by Aβ oligomers can trigger cell death [57–59].

**Fig. 1:**
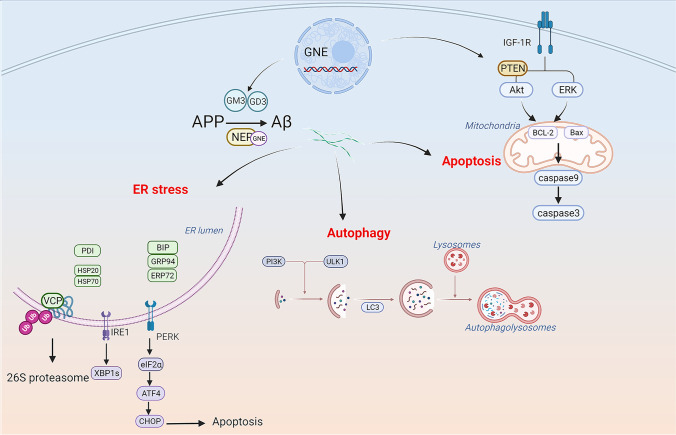
Molecular events present in GNE myopathy. Abnormal GM3 and GD3 lead to Aβ synthesis disorder in the Golgi apparatus and endoplasmic reticulum of GNE. At the same time, hyposialylated NEP cannot clear Aβ. Aβ deposition generates ER stress in GNE-mutant cells, which further triggers survival or apoptotic signaling mediated by IRE1-α or PERK, respectively. Molecular chaperones, ERAD, and UPS are all involved in the clearance of misfolded proteins. In the case of Aβ deposition, the autophagy-lysosome pathway is activated immediately to correct or degrade Aβ. Many molecules related to apoptosis in GNE myopathy, such as caspase 3, caspase 9, and IGF-1R, control cell survival and apoptosis by regulating the balance between AKT and ERK

SA is an essential monosaccharide in protein glycosylation, as it plays a vital role in protein folding. When its content decreases, it leads to abnormal folding of intramuscular proteins. Protein glycosylation includes N- and O-glycosylation [60]. GNE myopathy muscles show mainly O-hypoglycosylation [61]. It has been demonstrated that enhancing N- and O-glycosylation can reduce Aβ secretion [62, 63]. GNE can regulate sialyltransferase mRNA levels, thus influencing the cellular levels of GM3 and GD3 gangliosides, which are generated in the ER and play a role in regulating numerous cellular pathways [64, 65]. Abnormal GM3 and GD3 may affect Aβ generated from APP in the trans-Golgi network or ER [66] (Fig. 1). Low SA levels facilitate Aβ1-42 endocytosis, eventually leading to intracellular Aβ accumulation, which is regulated by clathrin-dependent endocytosis and heparan sulfate proteoglycans in the C2C12 myotube [67, 68]. Several enzymes can achieve Aβ peptide clearance, such as neprilysin (NEP) and insulin-degrading enzyme (IDE) [69–71]. NEP is an endopeptidase with multiple N-glycosylation sites and contains a large amount of SA [72]. In all GNE myopathy muscles, NEP is hyposialylated, with consequently reduced expression and enzymatic activity [69]. In abnormal muscle fibers of patients with GNE myopathy, Malicdan et al. found that the increase of Aβ in DMRV mice was related to the decrease in NEP activity, which could be reversed by treatment with tetra-O-acetyl- + N-acetylmannosamine (Ac4ManNAc) [73] (Fig. 1). In mouse models, disruption of the *NEP* gene consistently led to increased Aβ40 and Aβ42 levels and amyloid plaques in the brain [74]. Therefore, the low sialylation level in GNE myopathy results in an increased Aβ production and abnormal clearance, finally leading to deposition in muscle fibers. In the case of Aβ deposition, the protein quality control (PQC) pathway identifies abnormal proteins and corrects or degrades them to maintain homeostasis [75]. The PQC system contains molecular chaperones, the autophagy-lysosome pathway, and the ubiquitin–proteasome pathway.

### Aβ deposition, molecular chaperones, and the ubiquitin-proteasome system

The ER involves various cellular processes, including protein biosynthesis, folding, assembly, and maintaining Ca^2+^ homeostasis. ER stress (ERS) occurs when Ca^2+^ transport disorders occurred and unfolded or misfolded proteins accumulate in the ER lumen [76] (Fig. 1). The unfolded protein response (UPR) and ER-associated degradation (ERAD) are activated to protect cells against toxic proteins and alleviate ERS conditions. Specifically, three major transmembrane proteins are activated: transcription factor 6 (ATF6), inositol-requiring enzyme 1 (IRE1), and protein kinase R-like ER kinase (PERK) [77, 78]. Chaudhary et al. proved the existence of ERS in patients with GNE myopathy through the IRE1/XBP1 and PERK/eIF2α/ATF4/CHOP pathways.

Moreover, IGF-1 aids in reducing ERS and resisting the apoptosis of *GNE*-mutant cells, thus exerting a protective effect [79]. In the ER, peptides are correctly folded with the help of molecular chaperones, such as protein disulfide isomerase (PDI) and peptidyl propyl isomerase [80]. In cells with *GNE* mutations, downregulation of peroxiredoxin IV (PrdxIV), an ER-localized peroxiredoxin, affects PDI reoxidation. Moreover, alterations in PrdxIV can affect the redox state of ER and facilitate misfolding and protein aggregation in GNE myopathy [81]. Increased protein levels of ERS chaperones BiP (GRP78), GRP94, ERP72, calnexin, and calreticulin were found in GNE myopathy. The same study showed colocalization of UPR proteins with Aβ in the ER. Upregulation of valosin-containing protein (VCP) and linkers suggests that ERAD is also used to decrease aggregated misfolded proteins [22] (Fig. 1). Interestingly, compared to sporadic inclusion–body myositis (SIBM), Nogalska et al. did not find similar evidence of UPR induction in GNE myopathy muscle biopsies [82]. The reason for a shortage of ERS signals in GNE myopathy may be that different *GNE* mutations lead to different intracellular responses and cause different clinical phenotypes [82, 83]. Western blot analysis has shown increased HSP70 and HSP20 levels in GNE myopathy [84]. In the early stages of protein folding, HSP70 binds to the protein, inhibiting the formation of aggregates. If proper folding is impossible, HSP70 can degrade Aβ oligomers through the proteasome degradation pathway [56] (Fig. 1). The high expression of these molecules indicates that chaperones play an essential role in response to Aβ. However, non-eliminated amyloid will accumulate near the ER, disturb Ca^2+^ homeostasis, induce ERS, and activate molecular chaperones [85]. The resulting excessive protein accumulation and the further imbalance of Ca^2+^ homeostasis will make it impossible for UPR to repair cell damage and cause irreversible cell apoptosis.

The chaperone system is the first process to degrade or repair misfolded proteins, followed by the ubiquitin–proteasome system (UPS). The UPS comprises the ubiquitin-binding system and the 26S proteasome [86]. Misfolded proteins can easily aggregate and precipitate in cells, forming huge ubiquitinated insoluble polymers. These huge polymers cannot be easily hydrolyzed by the proteasome, affecting normal cell function and leading to cell death [87]. Strong ubiquitin antibody reactivity was observed in vacuolated and non-vacuolated fibers in GNE myopathy, which indicates that abnormal proteins are ubiquitinated and that the UPS cannot successfully degrade protein deposits [17] (Fig. 1). These phenomena are not accidental Aβ deposits in the muscles of patients with GNE myopathy; they elicit a strong response from the PQC system, which may contribute to disease development.

### Aβ deposition and autophagy

Autophagy is a fundamental physiological process in which intracellular lysosomes degrade dysfunctional proteins and cell organelles. Autophagy can be categorized into macroautophagy (called “autophagy” hereafter), chaperone-mediated autophagy, and microautophagy [88]. Through autophagy, cells can clear metabolic wastes and break them down into amino acids and fatty acids for recycling, which is essential for maintaining the homeostasis of the internal environment in cells and tissues. Autophagy undergoes several stages. The characteristic hallmark of autophagy is the autophagosome formed by the double-membrane structure wrapping the organelle and part of the cytoplasm. The formation of autophagosomes consists of three stages: nucleation, elongation, and lysosomal degradation. Initial vesicles come from various membrane sources, including the ER, Golgi apparatus, and mitochondria. After ULK1 is phosphorylated, it recruits various autophagy-related gene (Atg) proteins to the site of autophagosome formation. At the same time, ULK1 phosphorylates Beclin1 activates the formation of type III phosphatidylinositol-3-kinase (PI3K) and promotes phagophore nucleation. Then, pre-autophagosomes package autophagy substrates tend to form autophagosomes with the assistance of autophagy-related proteins such as light chain 3 (LC3). These autophagosomes combine with lysosomes to form autophagolysosomes and complete substrate degradation [89]. Mitochondria are known as “energy factories” and play a key role in maintaining the structural stability of muscle fibers. The maintenance of mitochondrial function comes from stabilizing the protein structure, a double-membrane organelle containing nearly 1000 proteins [90]. Life and death can be regulated through a variety of cellular regulatory processes, such as ATP production, maintenance of intracellular Ca^2+^ homeostasis, and reactive oxygen species (ROS) production. An imbalance between oxidants and antioxidants leads to abundant mitochondrial damage [91]. Mitophagy is a targeted autophagy process in which cells selectively remove dysfunctional or excessive mitochondria to maintain the integrity of the mitochondrial pool for cellular homeostasis [92, 93]. The PINK1/Parkin pathway is most closely associated with neuromuscular disease [94, 95]. When a large amount of ROS or abnormal protein deposition occurs, the mitochondrial membrane potential decreases, causing the surface receptor protein PINK1 to bind to the outer mitochondrial membrane (OMM). Parkin is attracted to the OMM, which induces ubiquitination and conformational changes in the OMM proteins to be recognized and bound by OPTN, p62, NDP52, and NBR1, which work together to complete mitochondrial autophagy [96]. Even a small error during protein folding may be harmful to cells in higher organisms. In the case of Aβ deposition, the autophagy-lysosome pathway immediately identifies abnormally folded proteins and then corrects or degrades them [75].

An accumulation of lysosomal-related proteins can be observed in RVs; however, healthy muscles contain few autophagy markers [97]. We consider this to be due to the activation of the lysosomal system in GNE myopathy muscles [16, 22] (Fig. 1). Protein aggregates can adversely affect the activity of lysosomal system components [98]. Aβ42 interacts with and destroys the lysosome membrane [99]. In addition, aquaporin-4 (AQP-4) expression is detected in the muscle fibers of a patient with GNE myopathy, especially at the RVs or their surrounding areas [100]. AQP-4 may be related to the lysosomal degradation system and muscle fiber degeneration. By detecting LC3 puncta using specific antibodies, impaired autophagy in retinal pigment epithelial cells induced by *GNE* mutations was reported [101]. These observations are consistent with the notion that GNE myopathy is closely associated with autophagy initiation and compromised autophagy [22, 102].

Conversely, autophagy disorder and Aβ production are causally and reciprocally related. Reduced Aβ and LC3 expression due to N-acetylcysteine treatment provide additional evidence regarding the relationship between redox imbalance and autophagy dysregulation [103–105]. On the other hand, the apoptosis mechanism is activated once Aβ-induced stress exceeds a certain intensity threshold or if autophagy cannot adapt to the injury. Apoptosis also inhibits autophagy because caspases digest several essential autophagy proteins [106]. Cells clear Aβ proteins through the autophagy-lysosome pathway; however, the relationship between Aβ and autophagy-lysosome pathway impairment remains unclear. In the future, controlling autophagy to eliminate Aβ deposition in GNE myopathy is worth exploring.

Ragged blue and COX-deficient fibers have been found in DMRV muscles. Mitochondrial proteomic changes in DMRV revealed 80 downregulated proteins, including antioxidant proteins [107]. Eisenberg also reported the upregulation of many mitochondrial genes encoding mitochondrial proteins like COX [108]. Mitochondrial degradation products were found in the structure of autophagosomes, suggesting that mitophagy also exists in GNE myopathy. Ultrastructural analysis revealed mitochondrial dysfunction in GNE myopathy, which resulted from the release of pro-apoptotic proteins, including cytochrome C [107]. Aβ42 can lead to the expression of the mitochondrial fission protein, reduce the fusion protein expression, promote ROS generation, and induce loss of mitochondrial membrane potential, which triggers mitophagy to remove damaged mitochondria [109]. Inhibiting COX2 function, for instance, could protect neurons and reduce Aβ accumulation in the neurons of Alzheimer’s disease transgenic mice [110]. Mitochondrial dysfunction plays an important role in muscle pathology, and its mechanism of action needs to be further explored. Different mutants exert different changes in mitochondrial morphology. For example, r-V572L-GNE and r-D176V-GNE showed more apparent changes in mitochondrial morphology than r-M712T-GNE, which mainly manifested as more obvious mitochondrial swelling [111]. In the future, the degree of mitochondrial changes in muscle cells of patients with different *GNE* mutations should be investigated as a potential cause of different phenotypes of GNE myopathy.

### Aβ deposition and cell apoptosis

Apoptosis refers to programmed cell death. This process actively removes aging and abnormal cells in the body. Apoptosis defects can lead to tumors and other cell death disorders, whereas excessive activation can lead to cytopenic diseases. Apoptosis includes two classic pathways that eventually activate the caspase cascade: the external (death receptor pathway) and the internal pathway (mitochondrial pathway) [112]. Apoptotic pathways may respond differently to different *GNE* mutations [111]. Aβ is sufficient to induce muscle fiber cell apoptosis in vivo and in vitro and can cause muscle fiber loss and muscle dysfunction in patients with SIBM [113]. Aβ-related apoptosis is also found in GNE myopathy, which may reflect downstream autophagy failure. In tissue samples from patients with GNE myopathy, caspase 3 activation and caspase 9 expression levels increased, especially in atrophic muscle fibers [67, 113]. Except for the caspase-dependent pathway, poly(ADP-ribose) polymerase–mediated nuclear fragmentation and DNA damage in HEK cells [111]. The mitochondrial membrane potential of myoblasts in patients with GNE myopathy was significantly reduced, Bax expression levels were increased, and anti-apoptotic Bcl-2 expression was decreased [67], indicating the activation of the mitochondrial apoptosis pathway. In a HEK cell–based model of GNE myopathy, mitochondria-dependent apoptosis and IGF-1R phosphorylation were observed, and IGF-1R hyposialylation led to AKT phosphorylation and downregulation of the ERK pathway, which may rescue apoptotic cell death of *GNE*-deficient cell lines through Bcl-2 [114]. Furthermore, the levels of activated PTEN proteins were elevated in all GNE myopathy-cultured myoblasts, which might lead to muscle loss and stimulation with insulin through activation of the PI3K/AKT pathway [115] (Fig. 1). However, SA supplementation could not deactivate the apoptotic response of cells, and the mitochondrial membrane remained depolarized. In addition, intracellular Aβ has been shown to impair AKT activation [116]. The balance between the ERK and AKT pathways may determine the fate of cells in terms of their survival or apoptosis. A previous study has suggested that apoptosis occurs via the ATF4-ATF3-CHOP pathway in *GNE*-knockout pancreatic cancer cells [117]. These studies show that various effector molecules targeting signal proteins in apoptotic pathways can aid in preventing cell apoptosis caused by Aβ accumulation and low SA, thereby preventing the progression of GNE myopathy (Fig. 1).

### Muscle atrophy

**Fig. 2:**
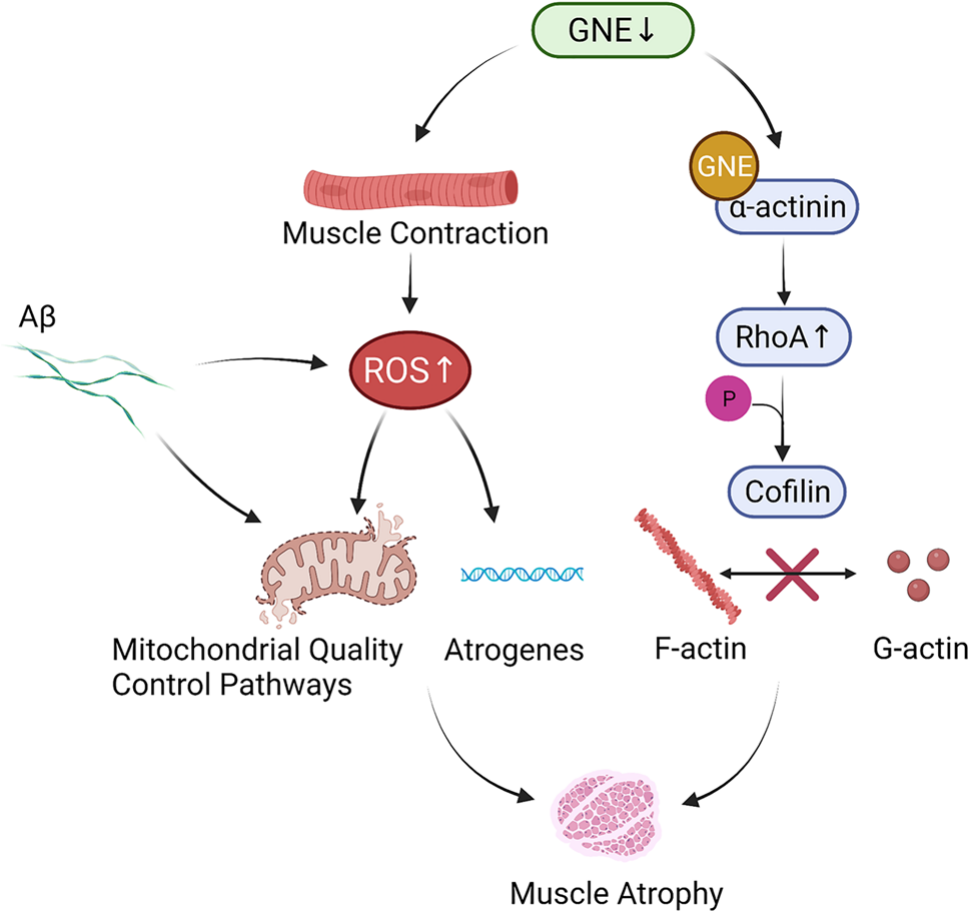
Molecular mechanisms of skeletal muscle atrophy. Mutations in the GNE gene lead to excessive ROS production after muscle contraction. At the same time, Aβ deposition also causes oxidative stress. Skeletal muscle mitochondria are disturbed and atrogenes are upregulated. GNE can interact with α-actinin and activate RhoA. Phosphorylation of cofilin hampers F-actin depolymerization and the generation of G-actin monomers. GNE gene mutation leads to cytoskeletal disruption and slowed cell migration. Consequently, muscle atrophy may occur

Muscle atrophy is most common when the protein degradation rate is higher than the synthesis rate. In a mouse model of GNE myopathy, Aβ deposition precedes muscle atrophy [17, 22]. Muscle contraction disorder is more severe after 40 weeks of age in GNE myopathy mice when RVs are formed. The oxidative stress induced by Aβ increases protein degradation in the skeletal muscles by triggering the UPS and molecular chaperones. If Aβ cannot be cleared, it will trigger autophagy, cell apoptosis, and mitophagy, thereby regulating and eventually affecting muscle mass [118]. Among all these processes, mitochondrial homeostasis plays a critical role in preventing muscle atrophy. Under protein deposition, the mitochondrial network accumulates damage, followed by oxidative stress. The production of mitochondrial ROS is the first step required to induce mitochondrial dysfunction and muscle atrophy [119, 120] (Fig. 2). Mitochondrial ROS can activate two main signal pathways, TGFβ/Smads and IGF1-AKT-mTOR-FoxO, to regulate muscle mass. Adenosine 5′-monophosphate (AMP)-activated protein kinase (AMPK) modulates FoxO3 transcriptional activity to induce muscle atrophy via activation of the UPS and autophagy pathways [121]. In addition to AMPK, PGC-1α and IGF/Akt/mTOR interfere with each other to regulate mitochondrial quality control, mitochondrial biogenesis, mitochondrial fusion, fission dynamics, and mitochondrial autophagy, which are all involved in muscle mass maintenance [104, 122, 123]. Activated PTEN and PDK1 proteins are important regulators of hypertrophy and atrophy of skeletal muscles, especially insulin action and glucose metabolism [115, 124, 125]. Stimulated PTEN expression has been shown to reduce muscle growth [126]. Hence, several unknown mechanisms control mitochondrial quality and the pathways that link mitochondrial dysfunction to muscle mass regulation in GNE myopathy (Fig. 2).

SA is a neuraminic acid present in both N- and O-acyl forms. It has been confirmed to be the most copious terminal glycoconjugate on monosaccharides; it plays vital functions in biological processes that involve recognition, such as cell–cell recognition and receptor-ligand interaction. The molecular mechanism of muscle atrophy in GNE myopathy is unclear. Yonekawa et al. found that muscle atrophy and weakness can be rescued by SA supplementation in GNE myopathy mice; Aβ and RVs almost disappeared in GNE myopathy mice with a high-dose 6′-sialyllactose [127]. SA deficiency in GNE myopathy muscles results in abnormal ROS production after muscle contraction. This increases oxidative stress in hyposialylated muscles, which causes upregulation of atrogenes, thereby leading to myofiber atrophy and enhancing S-nitrosyl modification of contractile and metabolic proteins. Dysregulation of autophagy exacerbates oxidative stress, which appears in the advanced stages of GNE myopathy. Notably, oral administration of N-acetylcysteine (an antioxidant) in *GNE*-mutant mice was shown to ameliorate muscle atrophy and autophagy [128]. Devi et al. developed a skeletal muscle cell–based model labeled GNE Exon‑3 Knockout Cell Line (SKM‑GNEHz). They found that GNE interacts stronger with α-actinin, which leads to RhoA activation and, in turn, phosphorylates cofilin. Increased cofilin phosphorylation hampers F-actin depolymerization, thus disturbing actin dynamics and generating G-actin monomers. Also, it may inhibit cell migration in SKM‑GNEHz cells, which is significant in muscle contraction and regeneration [129]. Similarly, Yadav et al. found that GNE is involved in regulating the actin assembly pathway through RhoA signaling [130] (Fig. 2). Previous studies indicated that GNE alters the β-1 integrin signaling response to fibronectin and influences α-actinin 1 and 2 [131–133]. These results suggest that *GNE* mutations influence cytoskeletal network proteins, which are important for muscle regeneration from satellite cells.

Moreover, SA directly contributes to changes in cell surface potential, and most voltage-gated channels are sialylated [134]. In rat skeletal muscles, hyposialylation of Na^+^ channels induces considerable changes in voltage-dependent gating of Na^+^ channels [134]. Therefore, the mechanism underlying this weakness may involve Na^+^ channel desialylation, resulting in decreased muscle membrane excitability. Protein sialylation homeostasis in hibernating Daurian ground squirrels’ skeletal muscles might protect them against disuse atrophy [135]. Similarly, increased resting Ca^2+^ and relative membrane depolarization in muscle fibers were found in a mouse overexpressing β-APP in type II fibers, which was related to clinical weakness [136]. In conclusion, a lack of sialylation may lead to skeletal muscle atrophy and weakness during the early stage of GNE myopathy. In later stages, Aβ deposition accelerates the oxidative stress response, resulting in increased free radicals and triggering mitophagy and apoptosis, thereby accelerating the progression of muscle atrophy.

## Conclusion and potential therapies

Currently, no therapy has been approved as a treatment for GNE myopathy. Because *GNE* mutations are the underlying cause of this disease, trials have focused on SA supplementation. Some studies confirmed that oral administration of monosaccharides and extended-release SA led to significantly higher levels of circulating free SA in patients with GNE myopathy [137, 138]. Niethamer et al. used a mouse model of GNE myopathy to show that potential sialylation-increasing monosaccharides effectively treat GNE myopathy [139]. Yonekawa et al. provided evidence that 6′-sialyllactose was more effective than free SA because it stayed longer in the body [127]. Unfortunately, the phase III clinical study on SA supplementation did not achieve the desired results. Therefore, SA supplementation alone may not be enough to recover the disease phenotype [140]. There are likely other therapeutic targets for GNE myopathy, and further research should focus on its pathological phenomena. In GNE myopathy, RVs may result from protein misfolding or aggregation clearing failures [141]. Intracellular protein deposition and RVs in the central part of muscle fibers may interfere with muscle force generation. N-acetylcysteine is a strong antioxidant, which improves the myopathy phenotype of GNE myopathy model mice by fighting the oxidative stress response caused by protein deposition [128]. The amyloid increase may be related to decreased NEP activity; Ac4ManNAc can reverse NEP activity and reduce Aβ deposition [73]. This may explain why early SA supplementation is effective, as it reduces Aβ production at an early stage [73]. In GNE myopathy, Aβ deposition triggers autophagy, and rapamycin is an inducer of autophagy, an mTOR inhibitor that can promote the clearance of protein aggregates and reduce the toxic effect of Aβ. It is unclear whether rapamycin can slow down Aβ deposition in GNE myopathy. Future treatment strategies could aim to hinder Aβ deposition from reducing disease progression.

GNE myopathy is caused by *GNE* mutations, which decrease SA content in skeletal muscle cells. In addition, cells with GNE myopathy show a characteristic Aβ accumulation. However, SA supplementation has not yielded ideal results. On the other hand, recent studies have revealed that aiming to increase Aβ clearance can serve as a treatment against neurodegenerative diseases; therefore, regulating Aβ production and elimination in GNE myopathy is meaningful. This review summarized the relationship between Aβ and GNE myopathy. Failure of Aβ clearance leads to the classic pathological phenomenon of RVs and muscle atrophy. Future studies should investigate ways to reduce Aβ deposition caused by the decreased enzymatic activity to treat GNE myopathy and intervene in the PQC systems to reduce the toxic effects of Aβ.

## Data Availability

Not applicable.
